# Alveolar Bone Box Ostectomy Grafted with Particulate Bone Substitute with Subsequent Dental Implant Placement in a Case of Craniofacial Fibrous Dysplasia Involving the Posterior Maxilla: Case Report and Literature Review

**DOI:** 10.3390/jcm12206452

**Published:** 2023-10-11

**Authors:** Fares Kablan

**Affiliations:** Department of Oral and Maxillofacial Surgery, Galilee College of Dental Sciences, Galilee Medical Center, Nahariya 2210001, Israel; kablanp1@gmail.com

**Keywords:** fibrous dysplasia of bone, dysplastic alveolar bone, sclerotic alveolar bone, bone grafts, dental implants

## Abstract

Background: Patients with dysplastic bone diseases, including fibrous dysplasia (FD), represent a particular challenge for placement of dental implants. This is due to structural bony changes that may compromise the bone blood supply and plasticity, thus potentially affecting the process of osseointegration. This case report describes a novel approach for dental-implant-based rehabilitation of the posterior maxilla affected by craniofacial fibrous dysplasia (CFD), with 7 years of treatment follow-up. Case presentation: A 35-year-old female patient was referred due to a suspected unidentified bone lesion affecting the left side of the maxilla. A clinical and radiographic diagnosis of fibrous dysplasia was confirmed through a wedge bone biopsy. Particulate bone substitute was packed into a box-shaped ostectomy area of the lesion in the affected maxillary alveolar ridge. This was followed by the placement of four implants 6 months post operation. The implants were successfully integrated, as confirmed by clinical examination over 7 years of follow up. Conclusion: this treatment approach may be considered as a predictable and efficient treatment modality for dental implant rehabilitation in patients with a variety of fibro-osseous lesions, including fibrous dysplasia, which affect the alveolar bone.

## 1. Introduction

Fibrous dysplasia (FD) is a benign bone disorder characterized by the replacement of normal bone tissue by fibro-osseous connective tissue [1]. Asymptomatic FD is usually observed as an incidental finding on routine radiographs and through computed radiography. FD can affect single (monostotic) or multiple (polyostotic) bones and can occur as an isolated condition (fibrous dysplasia disease) or as part of the McCune–Albright syndrome (MAS) [2,3], where it is usually accompanied by polyostotic involvement, hyper-functional endocrinopathies, and skin discoloration (café au-lait) [4]. Craniofacial fibrous dysplasia (CFD) involvement is found in 50% of polyostotic and 27% of monostotic forms [5]. In MAS, the craniofacial bones are involved in 90% of cases and the skull base in 95%, with temporal bone involvement in 70% of cases [4].

CFD has a slight female predilection, and most cases occur between the first and the third decades of life, with disease stabilization when the patient reaches skeletal maturity. The condition is almost twice as common in the maxilla as in the mandible, usually with unilateral lesions in the posterior region [2,5,6].

A diagnosis of CFD should be based on the clinical history and on physical, radiographic, and histopathological findings [7,8]. The classic radiographic appearance is described as “ground glass” or a grainy appearance of the trabecular bone with ill-defined borders. In early stages, the lesion is mixed-to-low-density and appears cystic rather than having the classical ground glass form [7,9]. Cystic degeneration into sarcoma is rare with an occurrence of less than 1% [10].

The partial or complete edentulism is best rehabilitated with dental implant therapy, which has demonstrated considerable success in modern dentistry [11,12]. However, patients with dysplastic bone diseases represent a particular challenge for the placement of dental implants since the associated structural bony changes may compromise bone blood supply and plasticity, thus potentially affecting the process of osseointegration. Therefore, most clinicians avoid the insertion of implants directly in the affected bone and usually utilize the vascularized/non-vascularized autologous bone grafts for a resected area in the jaws [13,14], or as one group have reported, a lesion site defect after excision that was grafted with a bone substitute and a long healing period [15]. Yet, there have only been two reports demonstrating successful direct implant insertion into dysplastic gnathic bone in both the mandible and maxilla. Bajwa et al. (2008) reported a case of a 32-year-old female with FD/MAS who underwent successful osseointegration and loading of dental implants in the maxilla and mandible. The patient was noted to be functional at 5 years—the longest follow-up reported to date [16]. Adnot et al. (2019) described a 2-year follow-up of the insertion of two dental implants in a 64-year-old female suffering from focal FD in her left mandible [17]. Based upon the literature, there is an unclear risk of implant failure, with some reports stating that bone healing and integration may occur more slowly secondary to the quality of the bone. Moreover, FD may present with variable features, and has a wide spectrum of clinical, radiographic, and histopathologic presentations [7]. Therefore, an individuation of gnathic FD lesions should be considered when dental implants are planned, for example, among those cases with a sclerotic type of FD.

Here, we report a case of a successful dental-implant-based rehabilitation of the posterior maxilla affected by asymptomatic-sclerotic-type CFD characterized by hyperdense bone. The case was followed up for 7 years, and the description is accompanied by a literature review and a discussion of the unique surgical approach.

## 2. Clinical Presentation

**Figure 1:**
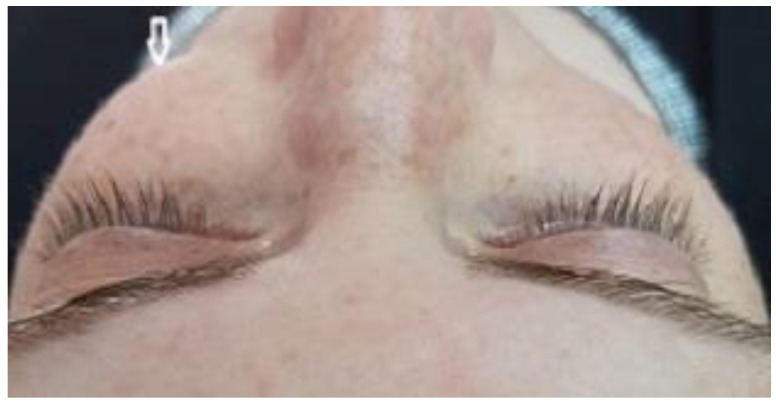
Clinical view of the patient shows mild facial asymmetry results from expansion of a CFD lesion in the left zygomaticomaxillary complex on the left side of the face (arrow).

**Figure 2:**
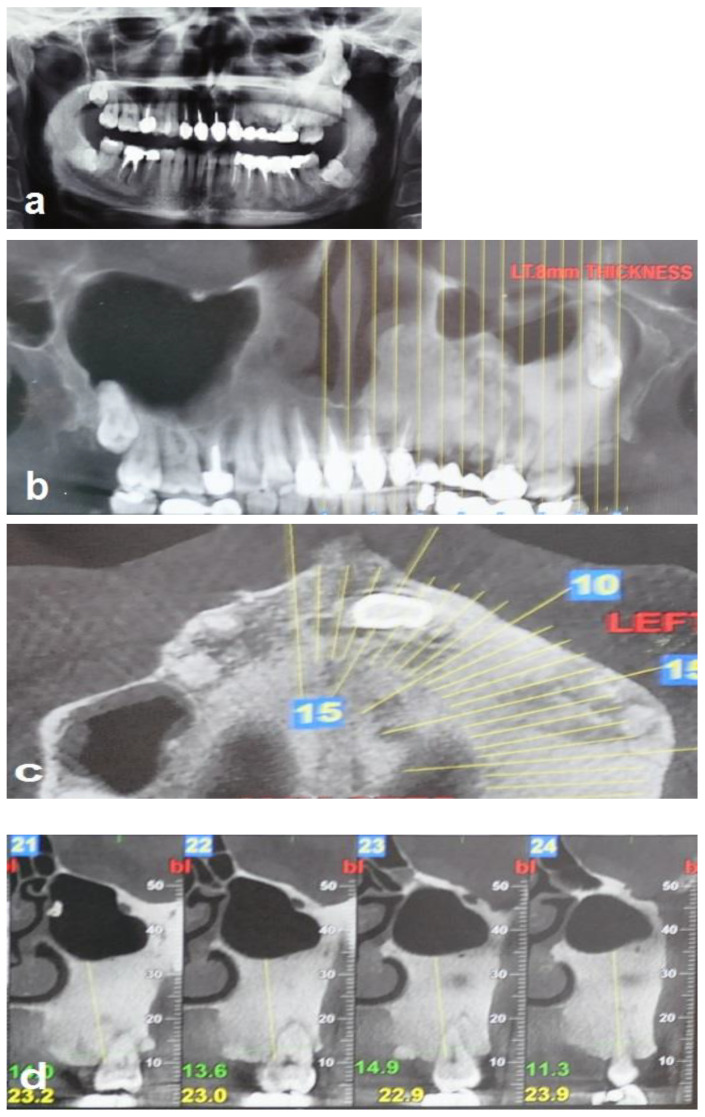
Radiographic views demonstrate that the FD lesion involves the left zygomaticomaxillary complex. (**a**,**b**) Panoramic radiograph and coronal CBCT show a hyperdense lesion in the left hemi-maxilla with displacement of the left maxillary sinus and the wisdom tooth, respectively. (**c**) Axial CBCT shows the hyperdense lesion involving the left maxilla, and (**d**) sagittal (cross-sectional) CBCT shows bone sclerosis and the displacement of the left maxillary sinus.

A 35-year-old woman was referred to the maxillofacial department for tooth extractions and dental implant placement in her left posterior maxillary segment. The referral was due to a suspected unidentified bone lesion in this region observed by her dental practitioner. She was an otherwise healthy patient. On examination, mild facial asymmetry was observed in her left malar area with mild expansion, without any neurological or cosmetic complaints (Figure 1). Intraoral examination revealed mild expansion of the vestibular left posterior maxillary ridge. An old fixed bridge extending from the left incisor to the first molar restored a missing canine, and first and second premolars. The first molar registered pain on percussion. Radiographic evaluation revealed a hyperdense “ground glass appearance” and sclerotic bone lesion affecting the left maxilla, left malar bone, and inferior and lateral orbital rims of the same side (Figure 2a–d).

**Figure 3:**
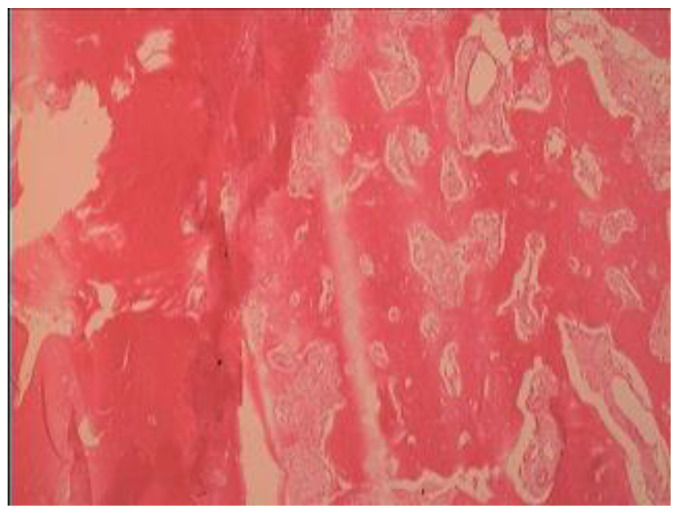
Histologic features of lesion (hematoxylin and eosin stain), relatively preserved continuity, and lamination of bony trabeculae. Hypocellular lesion with small amounts of fibrous tissue (alveolar bone ×400).

A clinical and radiographic diagnosis of fibrous dysplasia was confirmed through a wedge bone biopsy obtained on the day of the admission to the department (Figure 3). The obtained specimen from the lesion, fixed in formalin, was transported to pathologic examination. It was stained with a hematoxylin and eosin (H&E) after overnight decalcification using rapid decalcification solution containing hydrochloric acid. Treatment options that were discussed with the patient include en bloc resection of the involved alveolar ridge, reconstruction with a free iliac bone graft or fibular vascularized free flap, and subsequent implant placement. The patient refused both options. The treatment plan also included extraction of the first molar and placement of four implants. Due to the extreme density of the bone, and after detailed discussion with the patient, we decided to remove a box-shaped piece of the hyperdense bone from the maxillary ridge and replace it with particulate bone substitute. The implants will be subsequently placed as a second stage in the grafted new bone.

The patient was informed about the treatment modality and the biological rationale. In addition, the risks of inflammation and implant failure, and the need for long-term clinical and radiographic observation were discussed with the patient. After receiving and understanding this information, and after orally agreeing to the surgery, the patient’s written informed consent was obtained.

## 3. Surgical Procedures and Follow-Up

**Figure 4:**
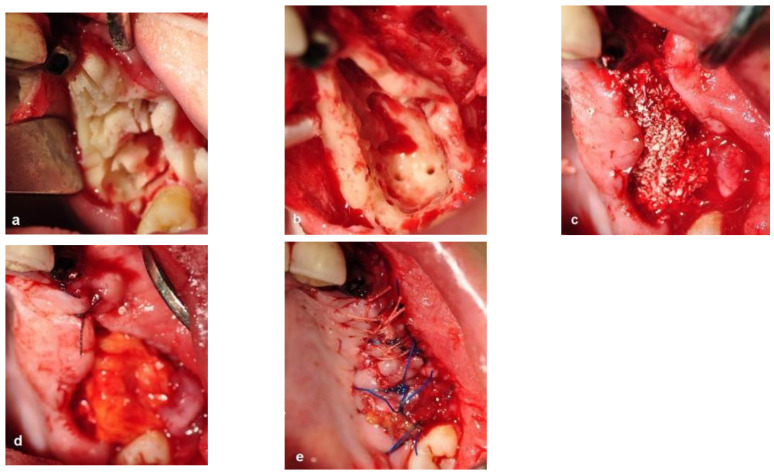
Intraoperative views. (**a**) Bone ostectomies in a box shape were carried out while preserving the buccal and palatal bone walls. No bleeding was observed. (**b**) Decortications of the box walls including the maxillary sinus bony floor were performed to enhance bleeding. (**c**) Grafting the box with particulate bone substitute. (**d**) PBFPG was utilized to enhance blood supply to the bone graft. (**e**) Primary closure of the flap with absorbable sutures.

The bone surgery was performed in the outpatient clinic under sterile conditions with intravenous sedation and local anesthesia. A midcrestal incision was performed at the edentulous ridge, with the envelope flap at the first molar. Anterior and posterior vertical releasing incisions were made, followed by buccal elevation of the flap. The first molar was removed. A box with dimensions of 2 cm anteroposterior and 1 cm bucco-palatal was outlined for bone evacuation, with the depth of the box limited by the floor of the maxillary sinus. Thin buccal and palatal walls were preserved. The bone ostectomies were performed with straight and round surgical burs. A marble bone quality without bleeding was noticed during the ostectomy (Figure 4a). The floor of the sinus was located 20 mm from the crest. Bleeding was induced by making small perforations in the bony floor of the maxillary sinus with a small round surgical bur (Figure 4b). Additional deep decortications were made in the palatal and buccal walls of the box. After completion of these procedures, particulate bone substitute (Bio-oss, Geistlich, Switzerland) was grafted and packed into the box (Figure 4c). Periosteal releasing cuts were made in the inner side of the mucoperiosteal flap in order to achieve soft tissue closure. In addition, a pedicled buccal fat pad graft (PBFPG) was obtained from the left buccal fat pad that was accessed through the distal vertical releasing incision. The PBFPG enhanced the soft tissue primary closure of the grafted box and served to provide blood to the compromised operated area (Figure 4d,e). Suturing of the treated site was performed using absorbable sutures (Vicryl 3/0, Peters Surgical, France).

**Figure 5:**
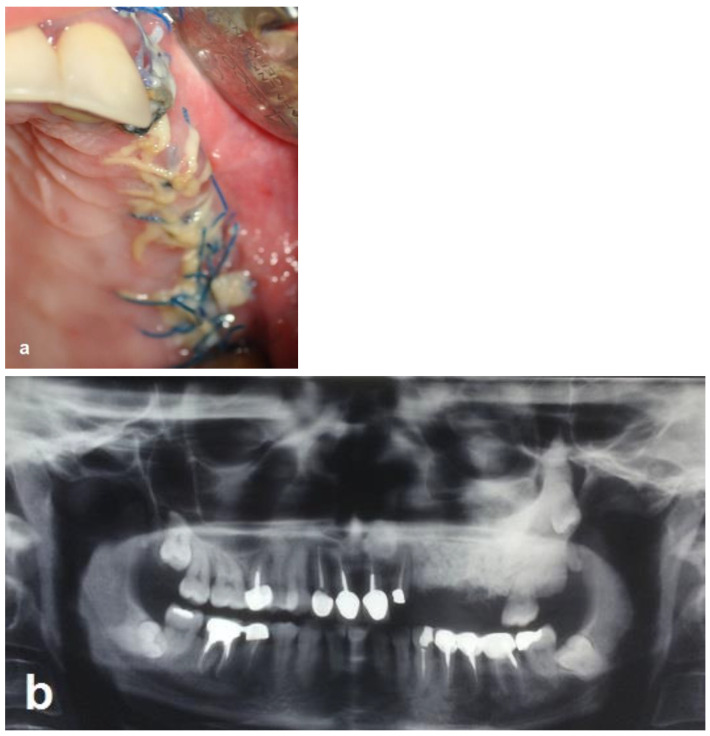
Follow-up 4 weeks after the operation. (**a**) Clinical view shows the maintenance of the primary closure without wound dehiscence. (**b**) Radiographic view shows the augmented area and the difference in opacity.

The patient was prescribed an antibiotic regimen (amoxicillin 1.5 g per day) for 14 days and was given post-operative oral hygiene instructions including mouth rinsing twice daily with chlorhexidine 0.12 for 2 weeks followed by topical application of chlorhexidine gel at the operated site. The patient was not permitted to wear a removable prosthesis until 6 weeks after the surgery. Four weeks after the surgery, the absorbable sutures were removed to enhance oral hygiene and a panoramic radiograph was obtained at this visit (Figure 5a,b).

**Figure 6:**
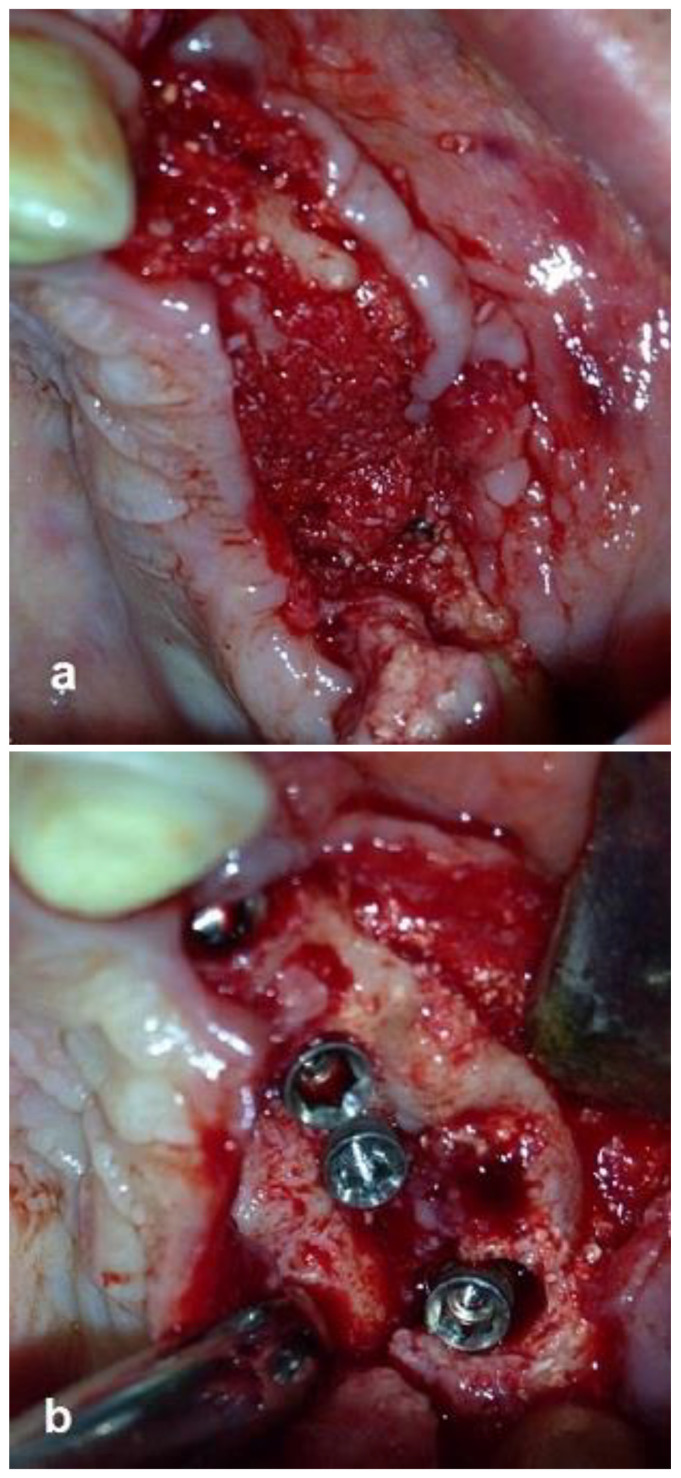
Re-entry after 6 months and insertion of implants. (**a**) New bone volume with obvious bleeding was demonstrated. (**b**) Placement of four implants in the new bone.

**Figure 7:**
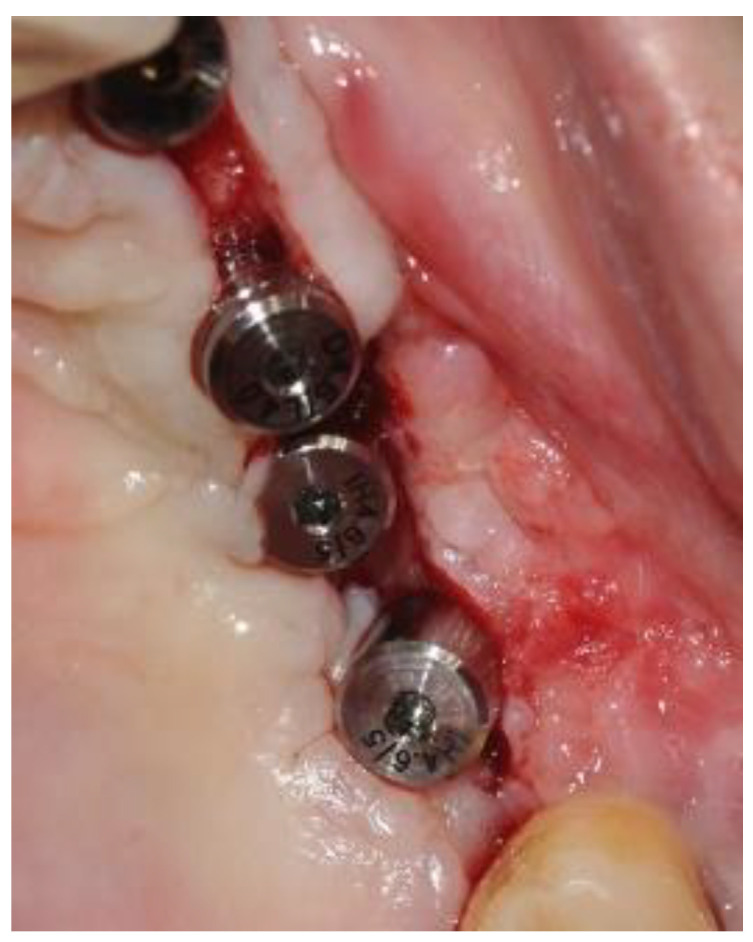
Implant exposure surgery and the insertion of four healing abutments. The image shows successful osteointegration of the implants.

**Figure 8:**
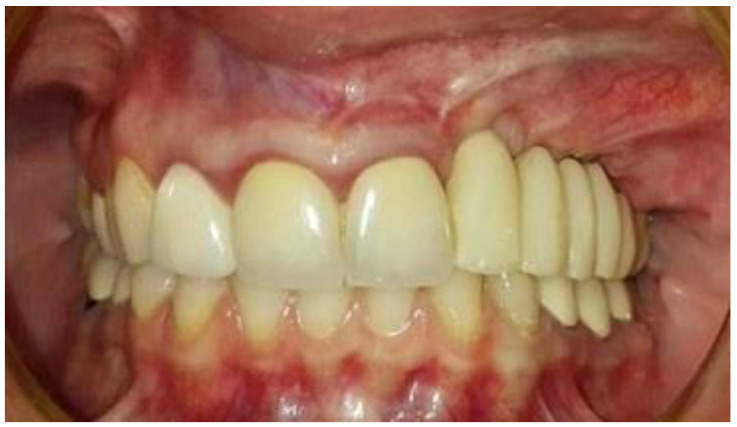
Temporary-fixed-prosthesis-supported dental implants. A good esthetical outcome is obvious.

**Figure 9:**
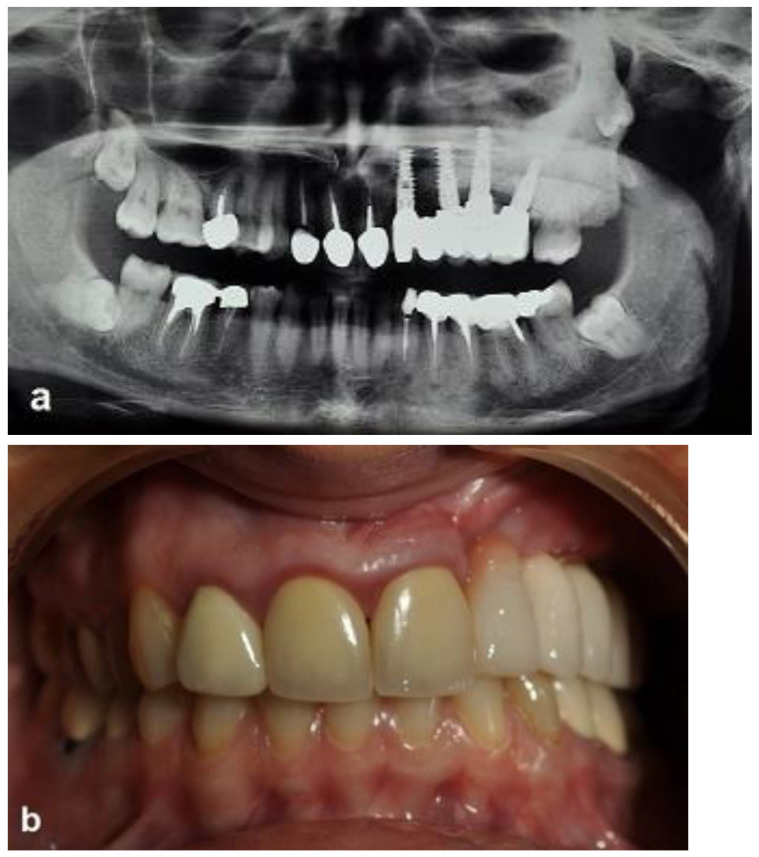
Ceramic-implant-supported fixed prosthesis one year after implant placement shows implant osseointegration and good aesthetics. (**a**) Radiographic view. (**b**) Clinical view.

The second-stage surgical intervention was carried out 6 months later for dental implant placement. Four implants (SPI-Alpha Bio Tec., Modi'in-Maccabim-Re'ut, Israel), 3.75 diameter/13 mm length, were placed in the new bone site in the left posterior maxilla (Figure 6b). The dental implants were uncovered 4 months thereafter (Figure 7). An acrylic bridge was fitted over the implants as a temporary prosthesis (Figure 8), and this was replaced 12 months later by a fixed ceramic prosthesis (Figure 9a,b). The patient was then followed over 7 years.

## 4. Results

Histology: Histologic features of the lesion (hematoxylin and eosin stain) showed a relatively preserved continuity and a lamination of bony trabeculae. In addition, it showed a hypocellular lesion with small amounts of fibrous tissue (alveolar bone ×100) (Figure 3).

**Figure 10:**
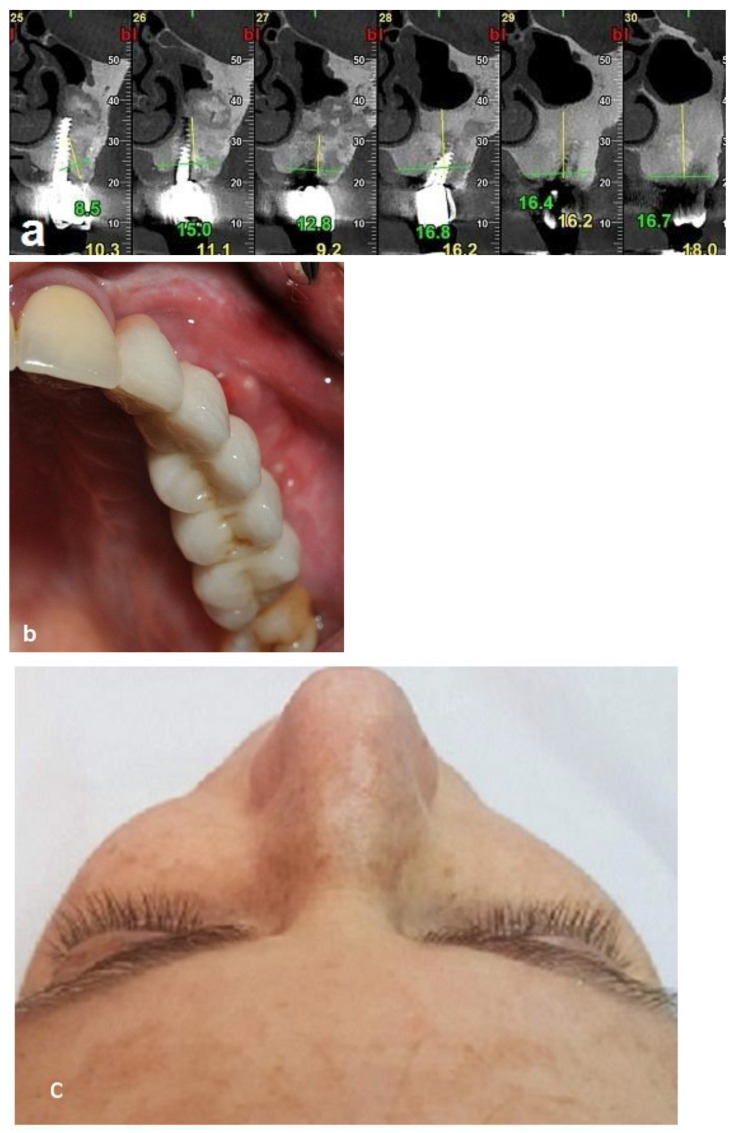
Clinical and radiographic images 7 years after the treatment show that there is no worsening of the FD lesion compared to the first picture that was obtained 7 years ago. (**a**) The CBCT view shows that the Bio-oss had been at least partially substituted by the FD lesion. (**b**) Intraoral view. (**c**) Extraoral views showing stable FD.

First surgery: Healing at the 4 weeks follow-up visit after the surgery was uneventful (Figure 5a,b). During dental implant placement surgery that was carried out six months after the first surgery, a good bone quality was observed with normal bleeding (Figure 6a). All implants were clinically and radiographically successfully osseointegrated when exposed 4 months later (Figure 7). The final rehabilitation after one year, and throughout the long period of the follow-up, provides satisfactory esthetic and functional outcomes (Figure 10a,b). There were no obvious changes in the lesion dimensions after 7 years (Figure 10c).

## 5. Discussion

Dental implants have long been used to rehabilitate edentulous and partially edentulous jaws, and have shown good long-term success [11,18]. However, patients with dysplastic bone diseases such as FD represent a unique challenge for oral rehabilitation with dental implants. This is because bone dysplasia is often associated with structural changes in the bone that compromise blood supply and plasticity, thereby causing potential problems for implant osseointegration. In addition, the abnormal bone development characteristic of the condition may complicate the implantation. The literature on dental implantology in FD patients is limited, and it is unclear whether there are significant problems in implant insertion and complications during and after the surgery. Moreover, fear of an increased risk of implant failure or risk of osteomyelitis in the setting of a failed implant may affect the insertion of dental implants in such cases [12,14]. An additional concern related to dental implant insertion in affected FD jaws is that the lack of osseous tissue and increased amount of fibrous tissue in some of the lesions may reduce the rigid fixation, thereby increasing the chances of local infection and spread of infection [12]. For these reasons, most clinicians avoid the direct insertion of implants into the affected bone, and rather favor vascularized/non-vascularized autologous bone grafts for a resected area in the jaws [13]. Of note is a case in which two implants were inserted in one patient, 10 months after the excision of fibrous dysplasia in the maxilla with bone substitute grafted to fill the gaps around the implants. The authors reported only one year of follow-up in that case [15].

A PubMed search revealed only two case reports describing successful direct implant insertion into dysplastic gnathic bone in patients with FD in both the mandible and maxilla [16,17]. Bajwa (2008) reported the insertion of nine implants in a patient with FD dysplasia affecting both jaws: five in the maxilla and four in the mandible. The implants were placed after conservative contouring of the affected areas. The authors reported that their patient was followed-up regularly for 5 years after the insertion of the implants, which remained well integrated by both clinical and radiographical criteria [16]. The second case report, presented by Adnot et al. (2019), described a 2-year follow-up of the insertion of two dental implants in a 64-year-old female suffering from focal FD in her left mandible. In this case, the outcomes on the affected side were comparable to synchronous dental implantation on the right side of her mandible [17]. To avoid the occurrence of submerged implants or revision of the prosthesis, there is a consensus that dental implants should be placed after a young patient has completed growth [12,19,20]. In addition, previous studies have shown that the radiographic appearance of FD changes with age. The homogeneous “ground-glass” appearance is most common during childhood and adolescence, whereas the lesions become less radiolucent, more mixed, and heterogeneous with age, with some older patients noted to have radiographically sclerotic lesions [19,20]. Taghsimi et al. (2022), in their narrative review, aimed to study the efficiency and safety of dental implantation in the area of hyperdense bone lesions, but only 19 articles matched the search criteria. The authors concluded that dental implants can be placed in some types of hyperdense jaw lesions. However, they stated that the possibility of complications and implant failure limits the use of dental implants in certain lesions like cement-osseous dysplasia. In addition, they suggested long-term clinical and radiological observation if the patient has undergone dental implantation [21].

Our case met the FD features reported by Davidova et al. (2020) [7] and includes the patient age (35 years), female patient, maxillary location, asymptomatic expansion, and ethnicity (Caucasian). The radiographic features of our patient exhibited ground glass opacity and sclerosis of the lesion. Histologically, cellularity of the lesion was sparse with hyperdense and sclerotic features of the affected bone.

As already noted, the unique histopathological characteristics of bone in CFD may hinder the success of directly implanted dental implants [16,17]. However, the variability in the histological and radiological appearances of CFD necessitates individualization of the treatment [7,20]. In our case, the highly opaque radiographic appearance of the lesion, and the hyper-osseous histopathological appearance of the lesion, suggested a hypo-vascularized bone tissue. This was obvious when surgically approaching the affected bone. There was no bleeding while cutting the bone, until the maxillary sinus floor was reached. In order to avoid insertion of implants into a bone with compromised vascularity, we first prepared a “box”, which was packed with particulate bone substitute in order to provide a better environment for subsequent dental implant insertion. Importantly there were no complications or infections of the surgical site, and radiographs revealed well-osseointegrated implants in the designated area in the maxilla. A 7-year follow-up demonstrates a stable disease, and successful functional oral rehabilitation of the inserted dental implants, without mobility, pain on function, or soft tissue inflammation.

Three reported cases in the literature used bone grafting in order to enhance dental placement in FD lesions. Monje et al. (2013) reported that a 49-year-old female patient presented an FD lesion on the left side of the maxilla. Their treatment included extraction of the involved teeth with simultaneous excision of the FD lesion, followed by insertion of two dental implants and simultaneous grafting of the cavity with an osteoconductive material after a healing period of 10 months. Both implants were successfully integrated, as confirmed by the clinical examination. The reported follow-up period of their case was one year [15]. Petrocelli et al. (2014) described the placement and 18-month follow-up of six dental implants in the mandible of a young patient, several years after bone augmentation from the calvaria and the iliac crest [14]. Di Carlo et al. (2019), in their case report, described the use of guided bone regeneration (GBR), to reconstruct maxilla in a patient with FD that was removed and reconstructed with a vascularized fibular graft and dental implant. Twelve years later, the onset of peri-implantitis led to the failure of osseointegration with consequent thinning of the fibula flap. The author described the use of GBR to avoid the risk of fracture and to restore the bone volumes necessary for a new implant-prosthetic rehabilitation. Two dental implants were placed 6 months after the augmentation surgery. A fixed implant-supported prosthesis with a custom-milled titanium bar screwed to the implants was made [22]. The difference between those three reports and our approach is that, in our treatment modality, the affected alveolar bone was locally excised and replaced with bone graft at the first surgical intervention, with subsequent placement of dental implants in the new and healthy bone as a second stage after a few months.

The main disadvantage of the “box ostectomy approach” is the need of second surgery to place dental implants including several months of waiting time for bone healing at the recipient site. However, it has several and significant advantages that include the following: 1. There is no need for partial/full resection of the jaw portion of the FD lesion, and reconstruction with non-vascularized/vascularized bone grafts, which is a treatment that is considered a major operation that requires prolonged hospitalization and a prolonged healing period. 2. Reducing post-operative morbidities and expense. 3. Can be carried out in outpatient clinic under local anesthesia or IV sedation, without hospitalization. 4. Minor surgical procedure, low complication rate, and insignificant morbidities. 5. Bone augmentation of the alveolar crest is a well-established procedure with high success rate. 6. Significant reduction in the treatment expense.

According to our experience with this case, we advocate this treatment modality for cases with dysplastic bone and cases with hyperdense bone of the edentulous area requiring dental implantation.

## 6. Conclusions

This case report presents a successful implant placement with a 7-year follow-up in a 35-year-old woman requiring fixed-restoration-supported dental implants. The presented approach of replacing the affected alveolar bone with a bone graft at the first surgical intervention, with subsequent placement of a dental implant in the new and healthy bone as the second stage, proved efficacious in treating this patient. The excision of dysplastic bone involving the alveolar ridge and its replacement with a bone graft is a very simple method, which can be used to introduce dental implants in individuals with fibrous dysplasia, and other bone conditions. We recommend a prolonged clinical and radiographic follow-up, with the patient maintaining good oral hygiene and attending periodic prophylaxis that includes oral hygienist treatment and reinforcement of the prosthesis screws.

## Data Availability

The required data can be obtained from the patient file at the private clinic. Fares Kablan kablanp1@gmail.com. The surgical file may be obtained from Baruch Pade Medical Center, Tiberias, Israel. File dates 2014–2015.
